# Neuromodulation and neuroprotective effects of chlorogenic acids in excitatory synapses of mouse hippocampal slices

**DOI:** 10.1038/s41598-021-89964-0

**Published:** 2021-05-18

**Authors:** Mara Yone D. Fernandes, Fernando Dobrachinski, Henrique B. Silva, João Pedro Lopes, Francisco Q. Gonçalves, Felix A. A. Soares, Lisiane O. Porciúncula, Geanne M. Andrade, Rodrigo A. Cunha, Angelo R. Tomé

**Affiliations:** 1 CNC-Center for Neuroscience and Cell Biology, University of Coimbra, Coimbra, Portugal; 2 Department of Physiology and Pharmacology, Faculty of Medicine, Center for Research and Drug Development (NPDM), Federal University of Ceará, Fortaleza, Brazil; 3 Centro de Ciências Naturais E Exatas, Departamento de Bioquímica E Biologia Molecular, Universidade Federal de Santa Maria, Santa Maria, RS Brazil; 4 Departamento de Bioquímica, Instituto de Ciências Básicas da Saúde, Universidade Federal Do Rio Grande Do Sul, Porto Alegre, RS Brazil; 5 Faculty of Medicine, University of Coimbra, Coimbra, Portugal; 6 Department of Life Sciences, Faculty of Sciences and Technology, University of Coimbra, Coimbra, Portugal

**Keywords:** Cellular neuroscience, Synaptic plasticity

## Abstract

The increased healthspan afforded by coffee intake provides novel opportunities to identify new therapeutic strategies. Caffeine has been proposed to afford benefits through adenosine A_2A_ receptors, which can control synaptic dysfunction underlying some brain disease. However, decaffeinated coffee and other main components of coffee such as chlorogenic acids, also attenuate brain dysfunction, although it is unknown if they control synaptic function. We now used electrophysiological recordings in mouse hippocampal slices to test if realistic concentrations of chlorogenic acids directly affect synaptic transmission and plasticity. 3-(3,4-dihydroxycinnamoyl)quinic acid (CA, 1–10 μM) and 5-O-(trans-3,4-dihydroxycinnamoyl)-D-quinic acid (NCA, 1–10 μM) were devoid of effect on synaptic transmission, paired-pulse facilitation or long-term potentiation (LTP) and long-term depression (LTD) in Schaffer collaterals-CA1 pyramidal synapses. However, CA and NCA increased the recovery of synaptic transmission upon re-oxygenation following 7 min of oxygen/glucose deprivation, an in vitro ischemia model. Also, CA and NCA attenuated the shift of LTD into LTP observed in hippocampal slices from animals with hippocampal-dependent memory deterioration after exposure to β-amyloid 1–42 (2 nmol, icv), in the context of Alzheimer’s disease. These findings show that chlorogenic acids do not directly affect synaptic transmission and plasticity but can indirectly affect other cellular targets to correct synaptic dysfunction. Unraveling the molecular mechanisms of action of chlorogenic acids will allow the design of hitherto unrecognized novel neuroprotective strategies.

## Introduction

The regular consumption of moderate amounts of coffee affords a robust protection against age-associated chronic diseases^1–3^, thus increasing healthspan on ageing^4^. Brain diseases are a major burden of disease^5^ and coffee intake dampens neuropsychiatric diseases with a robust inverse relation between coffee intake and mood-related depressive conditions^6–8^ as well as with cognitive deterioration^9–11^. Caffeine is a major constituent of coffee involved in these neuroprotective effects^12^, especially since animal studies have identified that caffeine and selective blockade of the molecular targets of caffeine—adenosine A_2A_ receptors^13,14^—afford a robust protection against mood and memory dysfunction in animal models of brain disease^15^. These protective effects of caffeine against brain dysfunction, have been linked to the ability of caffeine and A_2A_ receptor antagonists to fine-tune the function of synapses, namely controlling synaptic plasticity and preventing synaptotoxicity^16–18^, a process which is at the core of the onset of depression^19^ and memory-related dysfunction such as Alzheimer’s disease^20^.

Importantly, coffee has a diversity of other bioactive constituents apart from caffeine, amongst which stem chlorogenic acids^21^. As most polyphenols, chlorogenic acids have been described as potent antioxidants^22^ and several studies have shown an ability of chlorogenic acids to affect brain function^23–27^. Thus, interventions with diets enriched in chlorogenic acids ameliorate mood^22,23^ and cognitive function^25,26^ in volunteers. Moreover, mounting evidence indicates that the intake of chlorogenic acids decreases the incidence of different brain diseases, such as dementia^27–30^, depression^31,32^ or brain ischemia^33–36^. Additionally, cellular studies demonstrated cytoprotective and neuroprotective effect of chlorogenic acids^28,37–41^. However, it has not yet been tested if chlorogenic acids can directly affect synaptic function and/or attenuate synaptic dysfunction, which occurs at the onset of brain diseases.

With particular care to select realistic concentrations of chlorogenic acids, we now used electrophysiological approaches to probe the ability of two different chlorogenic acids abundant in coffee beverages to affect synaptic transmission and plasticity in neuronal circuits of hippocampal slices, which are frequently used as representative neuronal circuits. Furthermore, hippocampal circuits are modified under conditions of mood and memory dysfunction, allowing the assessment of possible effects of chlorogenic acids on synaptic deterioration in experimental conditions modelling ischemia or Alzheimer’s disease.

## Results

### Effect of chlorogenic acids on synaptic transmission and plasticity

We first tested the effects of two different chlorogenic acids that are known to be abundantly present in coffee beverages on synaptic transmission and plasticity under physiological-like conditions. We used hippocampal slices to isolate putative direct effects on synaptic function and we tested ‘realistic’ concentrations of chlorogenic acids (1–10 µM), within the reported concentrations of chlorogenic acids reached in the plasma, that are similar to these measured in the brain parenchyma^42^ of volunteers ingesting chlorogenic acids in amounts equivalent to those found in coffee^43,44^.

**Figure 1:**
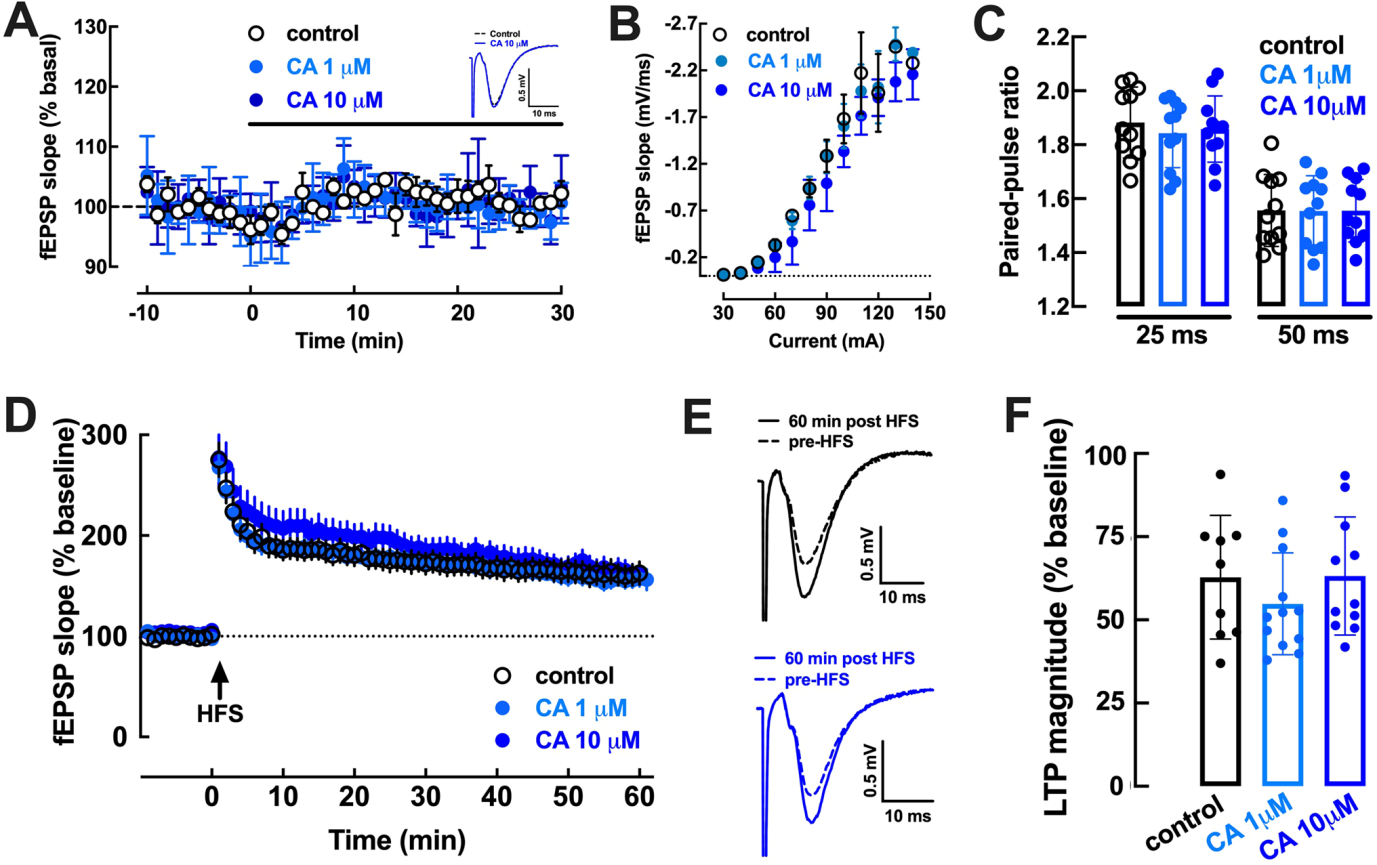
The chlorogenic acid 3-(3,4-dihydroxycinnamoyl)quinic acid (CA, 1 or 10 µM) did not affect either basal synaptic transmission or synaptic plasticity. CA (1 µM, light blue symbols or 10 µM, dark blue symbols) did not alter basal synaptic transmission (**A**), measured as the descending slope of field excitatory postsynaptic potentials (fEPSP) recorded extracellularly in the *stratum radiatum* of the CA1 area upon stimulation of the afferent Schaffer collaterals, illustrated as inserts in panel A. Likewise, CA (1 or 10 µM) did not modify the input–output responses (**B**) or the paired-pulse facilitation ratio, i.e. the ratio of the fEPSP slopes evoked by two consecutive pulses (ratio of P2/P1) with inter-pulse intervals of 25 or 50 ms (**C**). The average time course experiments of LTP induction (**D**), *i.e.* the variation of fEPSP slope over time upon induction of LTP with a high-frequency stimulation train (HFS: one train of 100 pulses of delivered at 100 Hz) in hippocampal slices untreated (control, black symbols) or treated with CA (1 or 10 µM), revealed that LTP magnitude was not affected by CA (**F**). (**E**) Representative fEPSP recorded in ACSF (control conditions, upper row) and in the presence of 10 µM CA (lower row) before (dashed traces) and 60 min after the HFS (filled traces). Data are mean ± S.E.M. of 6–12 experiments.

As shown in Fig. 1A,B, the superfusion of hippocampal slices with 3-(3,4-dihydroxycinnamoyl)quinic acid (CA, 1 or 10 µM) did not affect basal excitatory synaptic transmission (-1.18 ± 0.43% and 0.60 ± 0.95% modification of fEPSP slope; p = 0.094 and p = 0.999 *versus* control, n = 6, Wilcoxon Signed Rank test). CA (1–10 µM) was also devoid of effects on the paired-pulse facilitation ratio, with an inter-pulse interval of 25 ms or 50 ms, indicating a lack of direct presynaptic effects (Fig. 1C). Moreover, CA (1–10 µM) did not affect the magnitude of long-term potentiation, LTP (62.81 ± 6.19% over baseline under control conditions without drugs, 54.83 ± 4.42% over baseline with 1 µM CA and 63.22 ± 5.36% over baseline with 10 µM CA; n = 9–12, p = 0.467 *versus* control, Kruskal Wallis test) (Fig. 1D–F).

**Figure 2:**
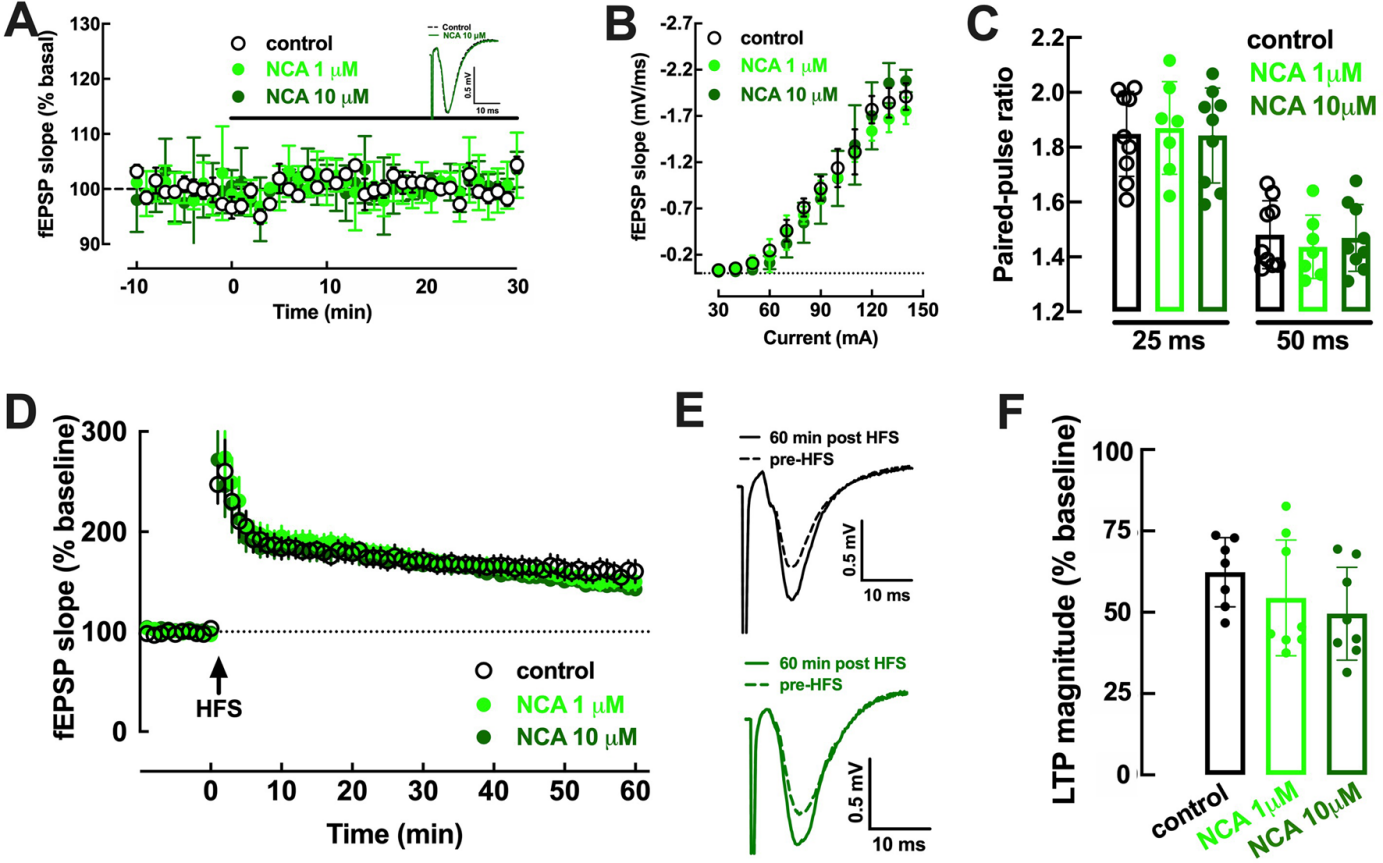
The neochlorogenic acid 5-O-(trans-3,4-dihydroxycinnamoyl)-D-quinic acid (NCA, 1 or 10 µM) did not affect either basal synaptic transmission or synaptic plasticity. NCA (1 µM, light green symbols or 10 µM, dark green symbols) did not alter basal synaptic transmission (**A**), measured as the descending slope of field excitatory postsynaptic potentials (fEPSP) recorded extracellularly in the *stratum radiatum* of the CA1 area upon stimulation of the afferent Schaffer collaterals, illustrated as inserts in panel A. Likewise, NCA (1 or 10 µM) did not modify the input–output responses (**B**) or the paired-pulse facilitation ratio, i.e. the ratio of the fEPSP slopes evoked by two consecutive pulses (ratio of P2/P1) with inter-pulse intervals of 25 or 50 ms (**C**). The average time course experiments of LTP induction (**D**), *i.e.* the variation of fEPSP slope over time upon induction of LTP with a high-frequency stimulation train (HFS: one train of 100 pulses delivered at 100 Hz) in hippocampal slices untreated (control, black symbols) or treated with NCA (1 or 10 µM), revealed that LTP magnitude was not affected by NCA (**F**). (**E**) Representative fEPSP recorded in ACSF (control conditions, upper row) and in the presence of 10 µM NCA (lower row) before (dashed traces) and 60 min after the HFS (filled traces). Data are mean ± S.E.M. of 4–8 experiments.

As shown in Fig. 2A,B, the superfusion of hippocampal slices with 5-O-(trans-3,4-dihydroxycinnamoyl)-D-quinic acid (neochlorogenic acid, NCA, 1 or 10 µM) did not affect basal excitatory synaptic transmission (0.80 ± 1.18% and 1.41 ± 0.78% modification of fEPSP slope; p = 0.438 and p = 0.250 *versus* control, n = 4–5, Wilcoxon Signed Rank test). NCA (1–10 µM) was also devoid of effects on the paired-pulse facilitation ratio, with an inter-pulse interval of 25 ms or 50 ms, indicating a lack of direct presynaptic effects (Fig. 2C). Also, NCA (1–10 µM) did not affect LTP magnitude (62.33 ± 4.04% over baseline under control conditions without drugs, 54.42 ± 6.29% over baseline with 1 µM NCA and 49.61 ± 5.04% over baseline with 10 µM NCA; n = 7–8, p = 0.204 *versus* control, Kruskal Wallis test) (Fig. 2D–F).

Overall, these findings indicate a lack of direct effects of chlorogenic acids on synaptic function under physiological conditions.

### Effect of chlorogenic acids on ischemia-induced depression of synaptic transmission

To test if chlorogenic acids could prevent the deterioration of synaptic function, we next resorted to an in vitro model of ischemia-induced depression of synaptic transmission. Ischemia was modelled by exposing hippocampal slices to oxygen and glucose deprivation (OGD^45,46^). Chlorogenic acids were applied at the onset of the re-oxygenation period since our previous studies indicated an efficiency of polyphenolic compounds to prevent ischemia-induced brain damage when applied after the onset of ischemia^47^. This approach offers the opportunity to clearly distinguish putative effects exerted by chlorogenic acids from these of caffeine, which mostly affects the onset of ODG-induced depression of excitatory transmission in the hippocampus^48^.

**Figure 3:**
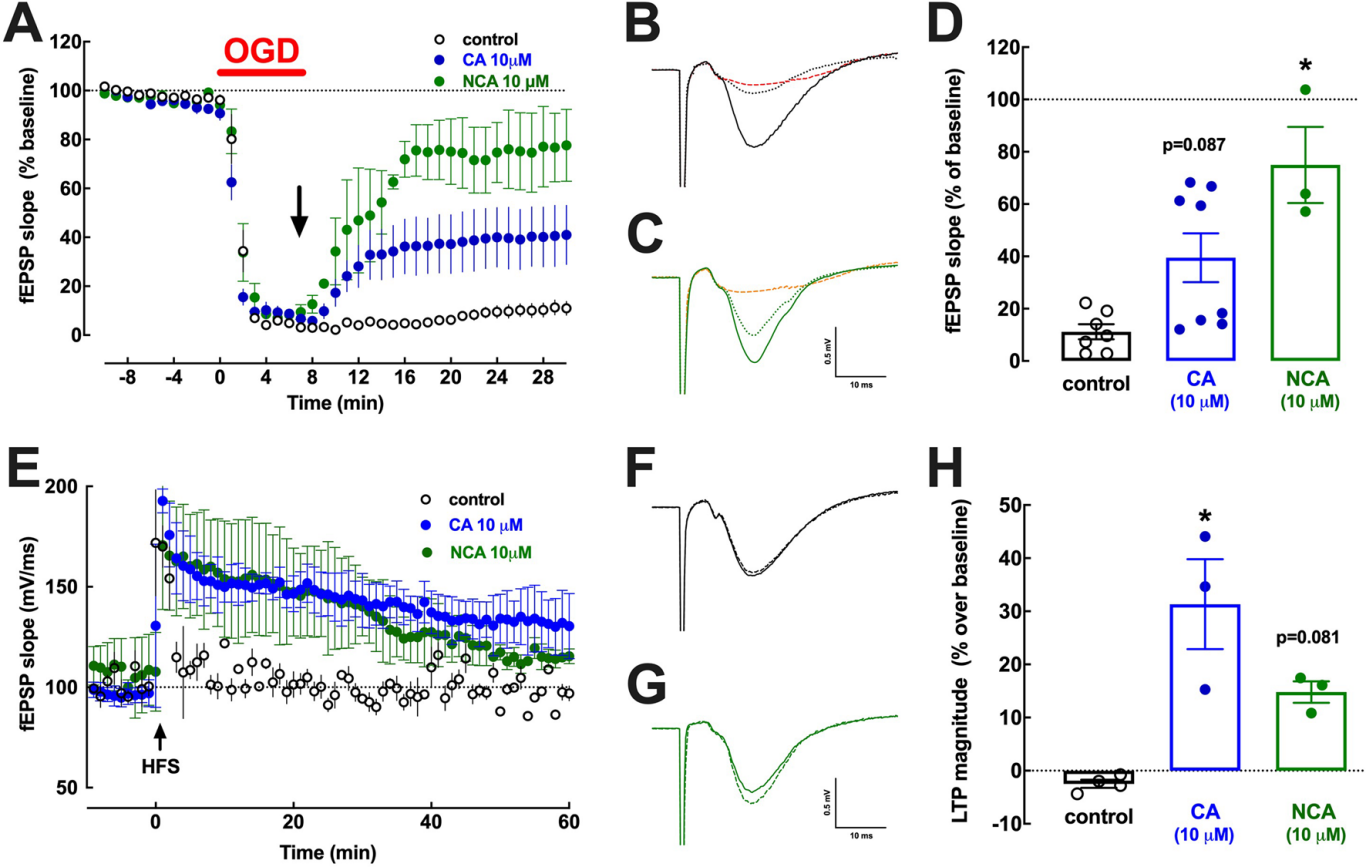
The chlorogenic acids 3-(3,4-dihydroxycinnamoyl)quinic acid (CA, 10 µM) and 5-O-(trans-3,4-dihydroxycinnamoyl)-D-quinic acid (NCA, 10 µM) accelerated and increased the extent of recovery of both hippocampal synaptic transmission and synaptic plasticity after exposure to oxygen–glucose deprivation (OGD), modelling ischemia. (**A**) A period of 7 min of OGD (red bar) caused a profound depression of hippocampal synaptic transmission, measured as the slope of field excitatory postsynaptic potentials (fEPSP) recorded extracellularly in the *stratum radiatum* of the CA1 area upon stimulation of the afferent Schaffer collaterals, without appreciable modification (< 15% around average) of the presynaptic volley amplitude throughout the whole protocol (data not shown). (**A**) Upon glucose and oxygen readmission (re-oxygenation), there was a discrete recovery of synaptic transmission in control conditions (no added drugs), whereas this recovery upon re-oxygenation was more robust in the presence of either CA (10 µM; blue symbols) or NCA (10 µM; green symbols), added when indicated by the arrow. (**B,C**) Representative superimposed fEPSPs before OGD (filled lines), 8 min after OGD (red/orange dashed lines) and upon reoxygenation, 24 min after OGD (dotted lines) in the absence (**B**) and in the presence of 10 µM NCA control conditions (**C**). (**D**) Quantification of fEPSP slopes between 20 and 25 min of re-oxygenation after OGD (**D**). The average time course experiments of LTP induction (**E**), *i.e.* the variation of fEPSP slope over time upon induction of LTP with a high-frequency stimulation train (HFS: one train of 100 pulses delivered at 100 Hz) in hippocampal slices subjected to OGD followed by re-oxygenation recovery revealed that LTP magnitude was discrete in slices not exposed to any drug (control, black symbols) but was more evident in slices treated with CA (10 µM) or NCA (10 µM) during re-oxygenation (**H**). (**F,G**) Representative superimposed fEPSPs before (filled lines) and 58 min after HFS (dotted lines) in the absence (**F**) and in the presence of 10 µM NCA control conditions (**G**). Data are mean ± S.E.M. of 3–8 experiments. * p < 0.05 (or exact p value) *versus* control using a Kruskal Wallis test followed by a Dunn’s post hoc multiple comparison test. Please note that the recordings from panels B and F are from the same slice in the absence of added drugs and the recordings from panels B and F are from the same slice in the presence of 10 uM NCA applied from reoxygenation onwards.

As shown in Fig. 3A, a 7 min period of OGD abolished synaptic transmission, which slightly recovered to 11.18 ± 2.89% of pre-OGD values (n = 7) within 20–25 min of re-oxygenation in control slices. Notably, the addition of 10 µM CA at the onset of re-oxygenation increased the recovery of synaptic transmission upon re-oxygenation to 39.49 ± 12.25% of pre-ischemic values (n = 8, p = 0.087 *versus* control, Dunn’s post hoc test after a Kruskal Wallis test) (Fig. 3A–D), although the data seemed to cluster into two distinct groups: some slices recovered markedly (n = 4 out of 8), whereas other slices recovered poorly (n = 4 out of 8). The addition of 10 µM NCA at the onset of re-oxygenation allowed a more evident increased the recovery of synaptic transmission upon re-oxygenation to 74.96 ± 14.53% of pre-OGD values (n = 3, p = 0.019 *versus* control, Dunn’s post hoc test after a Kruskal Wallis test) (Fig. 3A–D).

This increased recovery of synaptic function following OGD in the presence of chlorogenic acids was further confirmed by the greater ability of hippocampal synapses to implement patterns of synaptic plasticity in slices treated with chlorogenic acids after OGD. Thus, as shown in Fig. 3E (which is a continuation of the recordings displayed in Fig. 3A, re-scaled to facilitate comparisons), control slices subject to OGD were essentially unable to increase their synaptic efficiency after application of a high-frequency train (100 Hz for 1 s) normally able to trigger LTP (*e.g.* Figure 1D–F) (-2.47 ± 1.52% of baseline value, n = 4, p = 0.087 *versus* baseline). In contrast, slices treated with 10 µM CA from re-oxygenation onwards after OGD, were able to display LTP (31.3 ± 8.47% of baseline value, n = 3, p = 0.023 *versus* control, Dunn’s post hoc test after a Kruskal Wallis test) (Fig. 3E–H). Likewise, slices treated from re-oxygenation onwards after OGD in the presence of 10 µM NCA were also able to display LTP (14.8 ± 2.01% of baseline value, n = 3), although LTP magnitude was not significantly different from control (p = 0.081 *versus* control, Dunn’s post hoc test after a Kruskal Wallis test) (Fig. 3E–H).

In conclusion, in spite of the reduced number of experiments, the overall qualitative analysis of the findings suggests an ability of chlorogenic acids to recover synaptic deterioration in this in vitro model of ischemia.

### Effect of chlorogenic acids on alterations of synaptic plasticity in a model of Alzheimer’s disease

**Figure 4:**
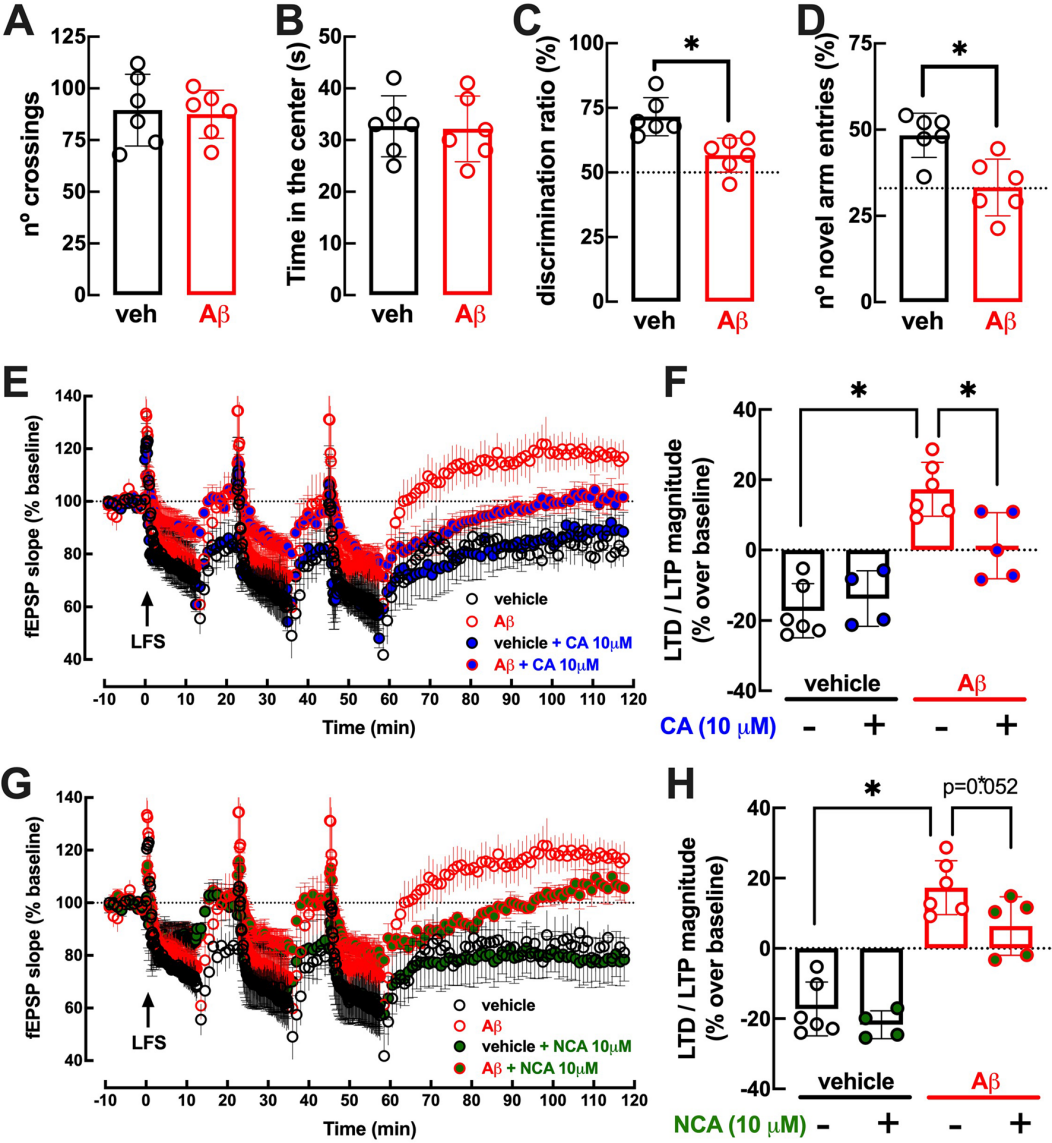
Mice injected intracerebroventricularly (icv) with β-amyloid peptide 1–42 (Aβ_1-42_) displayed a selective disruption of hippocampal-dependent memory performance, accompanied by a paradoxical alteration of hippocampal synaptic plasticity, which was attenuated by exposure to the chlorogenic acids 3-(3,4-dihydroxycinnamoyl)quinic acid (CA, 10 µM) and 5-O-(trans-3,4-dihydroxycinnamoyl)-D-quinic acid (NCA, 10 µM). Fifteen days after an icv challenge with Aβ_1-42_ (2 nmol), Aβ-treated mice (red symbols) displayed no alteration of spontaneous locomotion (**A**) assessed by the number of crossings in an open field test or anxiety, assessed as the time in the more aversive central area of the open field (**B**), compared with control (vehicle-treated mice; black symbols). In contrast, Aβ-treated mice displayed hippocampal-dependent memory deficits, as assessed in the object displacement test (**C**) and two-visits Y-maze test (**D**). Slices from vehicle-treated mice displayed a pattern of long-term depression (LTD) after repeated low-frequency stimulation (LFS), which was not affected by CA (10 µM; blue symbols) (**E–F**) or NCA (10 µM; green symbols) (**G,H**). Slices from Aβ-treated mice displayed a shift from LTD to LTP after repeated low-frequency stimulation (LFS), which was attenuated by CA (10 µM) (**E–F**) or NCA (10 µM) (**G,H**). Data are mean ± S.E.M. of 4–6 experiments. * p < 0.05 *versus* control using a Mann Whitney test (**C,D**) or a Kruskal Wallis test followed by a Dunn’s post hoc multiple comparison test (**F,H**).

Two weeks after the icv administration of 2 nmol Aβ_1-42_, there was no difference in the number of crossings between vehicle-treated (control: 89.5 ± 7.07, n = 6) and Aβ-treated mice (87.5 ± 4.75, n = 6; p = 0.937 *versus* control) in the open field test (Fig. 4A). This lack of altered locomotion was accompanied by an apparent lack of altered anxiety after Aβ exposure, as gauged by the similar time spent exploring the more aversive central area of the open field between vehicle-treated (control: 32.7 ± 2.40 s, n = 6) and Aβ-treated mice (32.2 ± 2.59 s, n = 6; p = 0.890 *versus* control) (Fig. 4B). In the object displacement test, the mice did not show a preference for the objects during training (data not shown), but Aβ-treated mice interacted less (p = 0.004) with the new object (56.7 ± 2.69% of total time, n = 6) than vehicle-treated mice (71.6 ± 3.00%, n = 6) (Fig. 4C). This Aβ-induced deterioration of hippocampal-dependent memory was confirmed using the modified Y maze: Aβ-treated mice entered less (p = 0.006) in the new and previously inaccessible arm (33.3 ± 3.36% of total entries, n = 6) than vehicle-treated mice (48.4 ± 2.62%, n = 6) (Fig. 4D).

In hippocampal slices from vehicle-treated mice, repeated trains of low-frequency stimulation (LFS) caused a long-term reduction of synaptic efficiency (Fig. 4E–F), corresponding to an LTD magnitude of -17.2 ± 3.14% (n = 6). LTD magnitude was not affected by 10 μM CA in slices from vehicle-treated mice (-13.8 ± 3.93%, n = 4; p = 0.909 *versus* control). In accordance with the previously reported shift from LTD-to-LTP associated with memory dysfunction^49^, LFS caused a LTP instead of a LTD in slices from Aβ-treated mice (17.3 ± 3.13%, n = 5), which decreased in magnitude in the presence of 10 μM CA (1.23 ± 4.21%, n = 6; p = 0.022 *versus* Aβ-treated) (Fig. 4E–F). Indeed, a Dunn’s test after a Kruskal–Wallis test indicated an effect of Aβ exposure in the absence of CA (p < 0.002) on the magnitude of LFS-induced synaptic plasticity, a difference which was no longer significant in the presence of 10 μM CA (p = 0.749).

Similarly, NCA (10 μM) also failed to alter LTD magnitude in slices from vehicle-treated (-13.8 ± 3.93%, n = 4; p = 0.780 *versus* control), whereas it attenuated the LTD-to-LTP shift in slices from Aβ-treated mice (6.36 ± 3.73%, n = 5; p = 0.052 *versus* Aβ-treated) (Fig. 4G–H). A Dunn’s test after a Kruskal–Wallis test indicated an effect of Aβ exposure in the absence of NCA (p < 0.011) on the magnitude of LFS-induced synaptic plasticity, a difference which was no longer significant in the presence of 10 μM NCA (p = 0.101). Please note that the data corresponding to control and Aβ-treated slices are the same for Figs. 4E–F and Figs. 4G–H.

These findings show that chlorogenic acids do not affect synaptic plasticity processes in ‘physiological’ conditions but recover synaptic deterioration in this model of Alzheimer’s disease.

## Discussion

The present study provides the first description of the putative direct effects of chlorogenic acids on synaptic function. The results obtained show that chlorogenic acids do not affect either hippocampal synaptic transmission or synaptic plasticity under physiological conditions; however, when the synaptic function is perturbed, either upon ischemia-like conditions or upon exposure to β-amyloid peptides, chlorogenic acids were able to attenuate synaptic dysfunction, by increasing the recovery of synaptic transmission on re-oxygenation or dampening the abnormal shift of hippocampal synaptic plasticity associated with memory impairment caused by exposure to β-amyloid peptides.

Chlorogenic acids are abundant in several everyday foods such as fruits and vegetables as well as in beverages, most prominently in coffee^21^. The positive impact of coffee intake on human health has been revived by the conclusion that coffee intake increases healthspan on ageing^1–4^ and is also inversely correlated with dysfunction of mood^6–8^ and memory^9–11^. Since mood and memory deterioration are now well established to involve modifications of synaptic function in afflicted brain areas^19,20^, it becomes particularly relevant to establish how different components of coffee beverages might affect synaptic function. Whereas a strong case has been made for a role of caffeine-mediated neuroprotection associated with the inhibition of synaptically-located adenosine A_2A_ receptor over-function^15^, decaffeinated coffee has also been reported to display health benefits^24,50^. This highlights the importance of defining the impact on the synaptic function of the most abundant chlorogenic acids present in coffee. The data presented in the present study show that concentrations of two different chlorogenic acids abundantly present in coffee and tested within the range estimated to be present in body fluids of individuals or animals exposed to doses of chlorogenic acids expected to be present in dietary components, namely coffee^42–44^, were devoid of direct effects on hippocampal synaptic transmission and plasticity. In fact, neither synaptic transmission nor input–output curves were affected by chlorogenic acids; also, short-term plasticity (paired-pulse facilitation) was unaffected, and there was no impact on the magnitude of either long-term potentiation (LTP) or long-term depression (LTD) in slices obtained from control mice (i.e. mice not exposed to any drug or perturbation expected to affect synaptic function). Altogether these observations show that chlorogenic acids are devoid of effects on synaptic transmission in physiological-like conditions.

In contrast to the lack of impact of chlorogenic acids on synaptic function under physiological conditions, chlorogenic acids alleviated the deterioration of synaptic function under pathology-like conditions. In fact, in slices obtained from A β-treated mice displaying a deterioration of hippocampal-dependent memory, we confirmed the presence of a shift from LTD into LTP following successive low-frequency stimulation trains^49^. Notably, whereas chlorogenic acids were devoid of effects in control slices they attenuated this abnormal shift from LTD to LTP in slices from Aβ-treated mice. This ability of chlorogenic acids to correct this putative neurophysiological trait of memory is in remarkable agreement with the ability of chlorogenic acids treatment to attenuate memory dysfunction in genetic^28,29^ or pharmacological models of Alzheimer’s disease^30^, as well as in patients with mild cognitive impairment^27^. Since in vitro studies show that chlorogenic acids can attenuate overall cellular toxicity upon exposure to either A β or glutamate in cultured neurons^37–40^ or neuron-like cell lines^41^, it is likely that the presently reported ability of chlorogenic acids to attenuate synaptic dysfunction in hippocampal slices from A β-treated mice might be an indirect consequence of a general cytoprotection afforded by chlorogenic acids rather than direct synaptic effects, in view of the previously discussed lack of direct effects of chlorogenic acids on synaptic function in ‘physiological’-like conditions.

A particular ability of chlorogenic acids to control synaptic dysfunction in pathological-like conditions was further confirmed by the observation that chlorogenic acids were able to accelerate the recovery of synaptic transmission upon re-oxygenation after oxygen–glucose deprivation, an in vitro model of ischemia^45,46^. Although with a limited number of experiments, the overall qualitative analysis of the data indicates that the exposure to chlorogenic acids during reoxygenation bolsters the recovery of synaptic transmission and the subsequent ability to engage synaptic plasticity phenomena. This is in accordance with the ability of chlorogenic acids to attenuate ischemia-induced brain damage in rodents^33–36^. These in vivo studies also showed that the treatment with chlorogenic acids ameliorated the performance in memory-related behavioral tests of the mice subject to brain ischemia^35,36^. Besides being in line with the proposal that hippocampal synaptic plasticity represents a neurophysiological basis of learning and memory processes, this is also in agreement with the presently observed larger magnitude of hippocampal synaptic plasticity in slices undergoing re-oxygenation in the presence of chlorogenic acids. However, this ability of chlorogenic acids to prevent the deterioration of synaptic function may result from an indirect effect of chlorogenic acids on glial cells supporting synaptic transmission rather than a direct effect on synaptic processes. In fact, it is well known that glial cells are affected by OGD challenges in hippocampal slices^51,52^ and that interfering with glial targets affects the sensitivity and recovery of synaptic function upon OGD^53,54^. Furthermore, chlorogenic acids can directly affect glial cells^31,55–58^ and control cell damage caused by oxidative stress in cell culture models^59–61^. Additionally, chlorogenic acids are devoid of effects on synaptic function in ‘physiological’-like conditions and the use of slices excludes the possible involvement of peripheral effects of chlorogenic acids that can indirectly impact brain function^62,63^. This strongly supports a new working hypothesis that CA might affect either metabolic or antioxidant processes in astrocytes^31^ or neuro-inflammatory-like reactivity in microglia^56^ to indirectly control synaptic dysfunction upon hypoxia. This would be in accordance with the ability of chlorogenic acids to control antioxidant and inflammatory processes related to glial cells in in vivo models of ischemia^33,34^. Finally, it is important to stress that, although the overall evaluation of the present findings is suggestive of a neuroprotective effect of chlorogenic acids in the recovery from OGD, the preliminary data raise some questions that will require additional experimental efforts, namely: (i) it is unclear why CA prompts a bimodal distribution of synaptic transmission after ODG, with some slices displaying a robust recovery whereas other recovered poorly; (ii) it is also unclear if a similar bimodal pattern would emerge if a larger number of experiments would be carried out with NCA; (iii) it is unclear if the apparent differences in the recovery of synaptic transmission and plasticity after OGD might result from different targets and/or different potencies of the different chlorogenic acids, an aspect that may be relevant for future selection of lead compounds for mechanistic and/or drug development studies.

In conclusion, the present study shows that chlorogenic acids only affect synaptic transmission and plasticity under pathological-like conditions, eventually through non-synaptic mechanisms. Since caffeine mostly affects synaptic transmission and plasticity controlling synaptotoxicity directly through its action on synaptically-located adenosine A_2A_ receptors^15^, the present findings that chlorogenic acids exert non-synaptic neuroprotective effects provides a rationale to propose possible complementary neuroprotective effects of caffeine and chlorogenic acids to underlie the robust neuroprotection afforded by coffee consumption. Future work designed to unveil the likely glial targets of chlorogenic acids may pave the way to develop future pharmacological strategies to alleviate the burden of brain diseases. Additionally, it will be important to detail if the present conclusions on the ability of chlorogenic acids to prevent alterations of synaptic function are valid for both sexes, since this preliminary study was carried out in male mice and there are compelling evidences indicating that some neuroprotective effects of coffee differ between men and women^64,65^.

## Materials and methods

### Animals

We used 37 male C57BL\6J mice with 10–12 weeks of age, obtained from Charles River (Barcelona, Spain). The use of only one sex of mice in this preliminary study was only dictated by their availability associated with the utilization of different tissues of the same animals by different research groups at our research Center. Animals were housed under controlled temperature (23 ± 2 °C), subject to a fixed 12 h light/dark cycle, with free access to food and water. Animal handling followed the ARRIVE guidelines and the European Community guidelines (EU Directive 2010/63) as approved by the Ethical Committee of the Center for Neuroscience and Cell Biology of Coimbra. All efforts were made to reduce the number of mice used and to minimize their discomfort. Thus, mice were anesthetized in halothane atmosphere before decapitation and, whereas the hippocampus was used in this study, other tissues were collected for other projects at our research center.

### Drugs

Chlorogenic acid (CA, 3-(3,4-dihydroxycinnamoyl)quinic acid; C3878 from Sigma, Sintra, Portugal) and neochlorogenic acid (NCA, 5-O-(trans-3,4-dihydroxycinnamoyl)-D-quinic acid; 94,419 from Sigma) were selected to be tested in this study since CA is the most abundant polyphenol in coffee beverages^21^ and we wished to confirm if any action of this main polyphenol was shared by one of its isomers also present in coffee, such as NCA^21^. CA and NCA were made up as 10 mM stock solutions in dimethylsulfoxide and directly diluted in ACSF to be applied to hippocampal slices, containing less than 0.01% dimethylsulphoxide that we have previously confirmed to be devoid of effects on synaptic transmission and plasticity^14^. Chlorogenic acids were tested at concentrations of 1 and 10 μM, similar to their estimated concentration in the brain and plasma upon ingestion of chlorogenic acids in amounts present in coffee beverages (0.6–14 μM)^42–44^.

### Electrophysiology

Following decapitation after halothane-induced anesthesia, the brain was quickly removed and placed in ice-cold, oxygenated (95% O_2_, 5% CO_2_) artificial cerebrospinal fluid (ACSF; in mM: 124.0 NaCl, 3.0 KCl, 1.25 Na_2_HPO_4_, 26.0 NaHCO_3_, 2.0 CaCl_2_, 1.0 MgCl_2_, 10.0 glucose). Slices (400 μm-thick) from the dorsal or ventromedial hippocampus were cut transverse to the long axis of the hippocampus and allowed to recover in oxygenated ACSF at room temperature for at least 1 h prior to recording, when they were transferred to a submerged chamber and superfused at 3 mL/min with oxygenated ACSF kept at 30.5 °C.

The extracellular recording of field excitatory post-synaptic potentials (fEPSP) was as previously described^14,16–18^ with a bipolar concentric electrode placed in the proximal CA1 *stratum radiatum* for stimulation of the Schaffer collaterals and the recording electrode, filled with 4 M NaCl (1–2 MΩ resistance), was placed in the CA1 *stratum radiatum* targeting the distal dendrites of pyramidal neurons. Stimulation was performed using either a Grass S44 or a Grass S48 square pulse stimulator (Grass Technologies, RI, USA), every 20 s with rectangular pulses of 0.1 ms. After amplification (ISO-80, World Precision Instruments, Hertfordshire, UK), the recordings were digitized (BNC-2110, National Instruments, Newbury, UK), averaged in groups of 3, and analyzed using the WinLTP version 2.10 software (WinLTP Ltd., Bristol, UK)^66^. The intensity of stimulation was chosen between 50 and 60% of maximal fEPSP response, determined on the basis of input/output curves in which the fEPSP slope was plotted *versus* stimulus intensity. Alterations of synaptic transmission were quantified as the % modification of the average value of the fEPSP slope taken from 15 to 20 min after the beginning of drug application, in relation to the average value of the fEPSP slope during the 5 min that preceded drug application through the superfusion solution.

The paired-pulse ratio was investigated by applying two pulses with an inter-pulse interval of 25 or 50 ms. Long-term potentiation (LTP) was induced by a high-frequency stimulation train (100 Hz for 1 s), as previously described^14,16–18^. LTP magnitude was quantified as the percentage change between the average slope of the five fEPSPs taken between 50 and 60 min after LTP induction in relation to the average slope of the fEPSP measured during the 10 min that preceded LTP induction. Long term depression (LTD) was induced as previously described^49,67^ in ventromedial hippocampal slices with 3 trains of 10 min stimulation at 2 Hz separated by 10-min intervals, with recordings performed at 32ºC. LTD magnitude was calculated as the percentage of change of fEPSP slope 50–60 min after completing LTD induction compared to baseline fEPSP (10 min before beginning LTD induction). The effect of drugs on synaptic plasticity was assessed by comparing LTP or LTD magnitude in the absence and presence of the drug in experiments carried out in different slices from the same animal.

### In vitro modelling of ischemia

The in vitro ischemia model consisted of a brief oxygen/glucose deprivation (OGD) applied directly to the superfused hippocampal slices, as previously described^45,46^. The OGD challenge consisted of replacing the oxygenated ACSF with an ACSF with 7 mM sucrose/3 mM glucose instead of 10 mM glucose and saturated with 95% N_2_/5% CO_2_, for 7 min, and thereafter resuming superfusion with the 10 mM glucose, oxygenated ACSF (re-oxygenation period). OGD causes a depression of synaptic transmission, which can recover totally or partially according to the duration of the OGD challenge^46^; we selected a duration of 7 min which caused a partial recovery of synaptic transmission on re-oxygenation, reaching values < 25% of the initial fEPSP magnitude in control slices (no added drugs), which was accompanied by a non-appreciable modification (< 15% around average) of the presynaptic volley amplitude throughout the protocol. When testing the effect of chlorogenic acids on OGD recovery, chlorogenic acids were only added on re-oxygenation onwards, i.e. they were absent prior to and during the OGD challenge; the recovery of synaptic transmission on re-oxygenation was quantified 20–25 min after re-oxygenation. The magnitude of the fEPSP slope was then adjusted to circa 50% of the initial fEPSP amplitude and the slices were subject to an LTP-induction protocol to assess LTP magnitude 60 min after the high-frequency train, as described above.

### Ex-vivo modelling of Alzheimer’s disease

Although there are no faithful animal models of Alzheimer’s disease (AD), intracerebroventricularly (icv)-injected Aβ_1-42_ might model early AD^68^, since it recapitulates the two main features of early AD, namely the impairment of reference memory and of synaptic function^18,68–70^. The Aβ_1-42_ peptide fragments (Bachem, Bubendorf, Germany) were dissolved in water to obtain a solution mostly composed of Aβ oligomers^68^ with a final concentration of 2.25 mg/mL. After anesthesia, mice were icv injected over a period of 15 min, with either a single dose of 2 nmol in 4 µL of Aβ_1-42_ or the same volume of water (vehicle, which caused no behavioral or neurochemical effects, similarly to the administration of scrambled Aβ_42-1_, see^68^). This apparently high dose of Aβ_1-42_ actually translates into 5–30 pmol levels of Aβ_1-42_ within the hippocampus, causing synaptic alterations and dysfunction^18,68^, without evidence of cellular damage^68^. Behavioral analysis was performed 14 days after Aβ_1-42_ or vehicle administration, at a time when spatial reference memory is selectively affected in this icv-Aβ_1-42_ model^68^. Behavior analysis was carried out as previously described^16,18^. Spontaneous locomotion was first monitored in an open-field apparatus. Hippocampal-dependent spatial reference memory was assessed with the object displacement test, using the same arena in a room (8 lx) with visual cues, where mice were first exposed to two identical objects for 3 min and 90 min later one of the objects was repositioned in the second session, where the interaction of mice with the objects was measured during 3 min. Hippocampal-dependent spatial reference memory was further assessed using a modified version of the Y maze test where we scored the number of entries and the time spent in the novel arm as a measure of spatial memory in a second 8 min visit to the maze 90 min after a first 8 min visit where one of the arms was inaccessible^16,18^. Mice were then sacrificed for preparation of hippocampal slices for electrophysiological recordings, as described above.

### Statistics

Values are presented as mean ± S.E.M. with the number of determinations (n, *i.e.* slices from different mice). The comparison of two experimental conditions was performed using a Mann Whitney test or a Wilcoxon Signed Rank test. Otherwise, statistical analysis was performed by a Kruskal Wallis test followed by a Dunn’s post hoc multiple comparison test. P < 0.05 was considered to represent statistical significance.

## Data Availability

The data that support the findings of this study are available from the corresponding author upon reasonable request.

## Abbreviations

A2AR: Adenosine A_2A_ receptor
Ab: β-amyloid peptide
ACSF: Artificial cerebrospinal fluid
CA: 3-(3,4-Dihydroxycinnamoyl)quinic acid
fEPSP: Field excitatory post-synaptic potential
icv: Intra-cerebroventricular
LTP: Long-term potentiation
LFS: Low frequency stimulation
LTD: Long-term depression
NCA: 5-O-(trans-3,4-dihydroxycinnamoyl)-D-quinic acid
OGD: Oxygen–glucose deprivation
